# Antibacterial Activity of THAM Trisphenylguanide against Methicillin-Resistant *Staphylococcus aureus*

**DOI:** 10.1371/journal.pone.0097742

**Published:** 2014-05-19

**Authors:** Alan J. Weaver, Joyce B. Shepard, Royce A. Wilkinson, Robert L. Watkins, Sarah K. Walton, Amanda R. Radke, Thomas J. Wright, Milat B. Awel, Catherine Cooper, Elizabeth Erikson, Mohamed E. Labib, Jovanka M. Voyich, Martin Teintze

**Affiliations:** 1 Department of Chemistry & Biochemistry, Montana State University, Bozeman, Montana, United States of America; 2 Department of Microbiology & Immunology, Montana State University, Bozeman, Montana, United States of America; 3 Advanced BioDevices, Princeton, New Jersey, United States of America; University Hospital Münster, Germany

## Abstract

This study investigated the potential antibacterial activity of three series of compounds synthesized from 12 linear and branched polyamines with 2–8 amino groups, which were substituted to produce the corresponding guanides, biguanides, or phenylguanides, against *Acinetobacter baumannii*, *Enterococcus faecalis*, *Escherichia coli*, *Pseudomonas aeruginosa* and *Staphylococcus aureus*. Antibacterial activity was measured for each compound by determining the minimum inhibitory concentration against the bacteria, and the toxicity towards mammalian cells was determined. The most effective compound, THAM trisphenylguanide, was studied in time-to-kill and cytoplasmic leakage assays against methicillin-resistant *Staphylococcus aureus* (MRSA, USA300) in comparison to chlorhexidine. Preliminary toxicity and MRSA challenge studies in mice were also conducted on this compound. THAM trisphenylguanide showed significant antibacterial activity (MIC ∼1 mg/L) and selectivity against MRSA relative to all the other bacteria examined. In time-to-kill assays it showed increased antimicrobial activity against MRSA versus chlorhexidine. It induced leakage of cytoplasmic content at concentrations that did not reduce cell viability, suggesting the mechanism of action may involve membrane disruption. Using an intraperitoneal mouse model of invasive MRSA disease, THAM trisphenylguanide reduced bacterial burden locally and in deeper tissues. This study has identified a novel guanide compound with selective microbicidal activity against *Staphylococcus aureus,* including a methicillin-resistant (MRSA) strain.

## Introduction

Ever since antibiotics were introduced into clinical practice, bacterial pathogens have been developing resistance which reduces or eliminates their effectiveness. In addition, opportunistic pathogens with innate resistance to antibiotics have become emerging problems, particularly in hospital settings. In developed countries, such as the United States, bacterial species that have acquired multiple drug resistance include the ESKAPE pathogens, aptly named for their ability to escape the effects of our current antimicrobial drugs. These include ***E*** *nterococcus faecium*, ***S*** *taphylococcus aureus*, ***K*** *lebsiella pneumoniae*, ***A*** *cinetobacter baumannii*, ***P*** *seudomonas aeruginosa* and ***E*** *nterobacter* species [1]. As an example, methicillin-resistant *Staphylococcus aureus* (MRSA) was once thought to be problematic only in healthcare settings, but now community-associated MRSA infections are becoming more common. MRSA have become resistant to the multiple classes of antibiotics including beta lactams, macrolides, and quinolones [2], as well as the glycopeptide vancomycin, the common drug of last resort [3].

We have synthesized three series of compounds, derived from linear and branched polyamines with two to eight amino groups, which were substituted to produce the corresponding guanides, biguanides, or phenylguanides [4]. Some of these compounds were previously found to be effective as antagonists of CXCR4 and inhibited HIV infection by X4 strains [4], [5] and the formation of metastatic tumors [6]. The biguanide series of compounds are chemically related to some commercially important biocidal agents found in common household items such as disinfectants, mouthwashes, contact lens solutions, and cosmetics. Examples include polyhexamethylene biguanide (PHMB), a heterogeneous polymer containing multiple positively charged biguanides, and the small-molecule bis-biguanides chlorhexidine and alexidine. Our new compounds are also polycationic, and preliminary experiments had shown low cytotoxicity *in vitro* of many of the new compounds [4]; we hypothesized that our compounds might have more selective antibacterial activity than these other biguanides. In the current study we investigate the antimicrobial potential of these compounds and demonstrate the efficacy of THAM trisphenylguanide against MRSA both *in vitro* and *in vivo*.

## Materials and Methods

### Guanide, Biguanide and Phenylguanide Compounds

The compounds were synthesized and purified as described previously [4], with the following additions: Trisoctylaminomelamine (TOAM) was synthesized using 1,8-diaminooctane as previously described for trishexylaminomelamine (THAM) except that an equal volume of acetonitrile was added to the reaction after 2 h in refluxing water to help with solubility of the more non-polar compound and the reaction was allowed to reflux for an additional hour in 1∶1 acetonitrile/water. The TOAM guanide derivatives were synthesized as described previously for the corresponding THAM compounds [4].

### Bacterial Strains

Bacterial strains used in these experiments were *Escherichia coli* (strain K91), *Pseudomonas aeruginosa* (strain PA01), *Acinetobacter baumannii* (strains ATCC 19606 and ATCC BAA1605), *Enterococcus faecalis* (strains MSU#10 and ATCC V583 [7]), and *Staphylococcus aureus* (strains RN4220 [8] and community-associated MRSA strain LAC, pulsed-field gel-electrophoresis type USA300 [9]). Three of the strains display known drug-resistant phenotypes. Specifically, *A. baumannii* BAA1605 is a multi-drug resistant isolate [10], [11], with resistance to ceftazidime, gentamicin, ticarcillin, piperacillin, aztreonam, cefepime, ciprofloxacin, imipenem, and meropenem. *E. faecalis* V583 is vancomycin and gentamicin resistant [7] and USA300 (strain LAC used in this study) is resistant to penicillin, oxacillin, erythromycin, and shows intermediate resistance to ciprofloxacin [9], [12], [13].

### Minimum Inhibitory Concentration and Minimum Bactericidal Concentration Assays

Minimum inhibitory concentration (MIC) values were determined for those compounds showing the most antibacterial activity in the bacterial viability assays (see Supplemental Materials) against each of the strains of bacteria using a microdilution method in 96 well plates with LB media according to guidelines published by the British Society for Antimicrobial Chemotherapy [14]. Minimum bactericidal concentrations (MBC) were determined by plating 10 µL and 100 µL from each well showing no signs of growth after overnight incubation of the MIC plates and determining the minimum concentration showing a 3-log reduction of viable bacteria. MIC and MBC were also determined in cation-adjusted Mueller-Hinton broth (Hardy Diagnostics, Santa Maria, CA) for some compounds and strains.

### Kill Curve Assay

Bacteria were cultured to mid-log phase (OD_600_ ∼0.4) in LB media with shaking and then diluted to approximately 10^4^ to 10^5^ CFU/mL. Bacteria were then treated with compound at the MIC, 2×MIC, and 4×MIC (as determined by the MIC assays). Over the course of 24 hours, bacteria were incubated at 37°C and aliquots removed at various time intervals, diluted, and plated on LB agar. Plated bacteria were incubated overnight at 37°C and CFU/mL were calculated for each time interval.

### Cytoplasmic Leakage & Bacteriolysis Assay

Bacteria were cultured to mid-log phase (OD_600_ ∼0.4) in LB media with shaking. Bacteria were then harvested and resuspended in PBS to a concentration of ∼10^8^ CFU/mL. Cultures were treated with compound at the MIC, 2×MIC, and 4×MIC and incubated at 37°C with shaking. Bacteria were monitored over time for cytoplasmic leakage via A_260_ measurements and for cell viability. For A_260_ readings, sample aliquots were centrifuged for 5 min at 10,000×g at room temperature to remove cellular debris. Viability was monitored by determining the CFU/mL during the first 3 hours post treatment as described above for the kill curve assays.

### Cell Toxicity Assays

Compound cytotoxicity was evaluated using the CellTiter 96® Aqueous Non-Radioactive Cell Proliferation Assay (MTS) following the manufacturer’s instructions (Promega, Madison, WI) using both the human breast cancer cell line MDA-MB-231 (ATCC HTB-26) and HaCaT human keratinocytes [15]. The HaCaT cell line was grown in Epilife medium (MEPI500Ca) with human keratinocyte growth supplement (Invitrogen, San Diego, CA). Both types of cells were harvested using Tryspin/EDTA and diluted to 1×10^5^ cells per well in a 96-well plate. Ten concentrations covering five orders of magnitude were tested with six replicates per concentration. Cells were incubated for 48–72 hours at 37°C with 5% CO_2_. MTS/PMS solution (Promega, Madison, WI) was added to the wells and absorbance was measured at 490 nm two to four hours later. Data was analyzed using GraphPad Prism version 5.0 (GraphPad Software, La Jolla, CA) using a variable slope inhibition dose response curve.

### In vivo MRSA Challenge Experiments

Three groups of BALB/c mice (8 mice per group) were infected by intraperitoneal (*i.p.)* injection with 5×10^7^ MRSA in 100 µl sterile PBS [16], [17]. The first treatment group was injected *i.p.* with 100 µl of 100 µM THAM trisphenylguanide in PBS immediately after infection. The second group was injected *i.p.* with 100 µl of 100 µM THAM trisphenylguanide one hour post-infection. The control group was injected with 100 µl PBS. At eight hours post-infection, all mice were euthanized. An intraperitoneal lavage using 10 ml PBS was performed to recover bacteria within the peritoneal cavity and plated at multiple dilutions on TSA plates. The heart and kidney were removed for evaluation of systemic infection. Briefly, tissues were weighed, homogenized, and plated at multiple dilutions on TSA plates. TSA plates were incubated overnight at 37°C at 5% CO_2_ and CFU enumerated the next day.

### Ethical Approval

All studies conformed to National Institutes of Health guidelines and were approved by the Animal Care and Use Committee at Montana State University (MSU)-Bozeman, MT (Protocol #2011-60).

### Statistics

Statistical analysis was performed using GraphPad Prism version 6.0 (GraphPad Software, La Jolla, CA). Data for each analysis represents a minimum of 2 repetitions. Data sets were analyzed using one-way analysis of variance with Bonferroni post-test or using Student’s t-test. Error bars represent the standard error of the mean.

## Results

### Antibacterial Activity *In vitro*

We initially screened all the guanide, biguanide, and phenylguanide compounds we had synthesized, and their parent amines, in a bacterial viability assay against three Gram- negative (*E. coli, P. aeruginosa,* and *A. baumannii*) as well as two Gram-positive species (*S. aureus* and *E. faecalis*). The IC_50_ values (Table S1) were used to estimate the relative activity level of the various compounds and determine which would be investigated further. Chlorhexidine was used as a positive control with known bactericidal activity [18].

**Figure 1:**
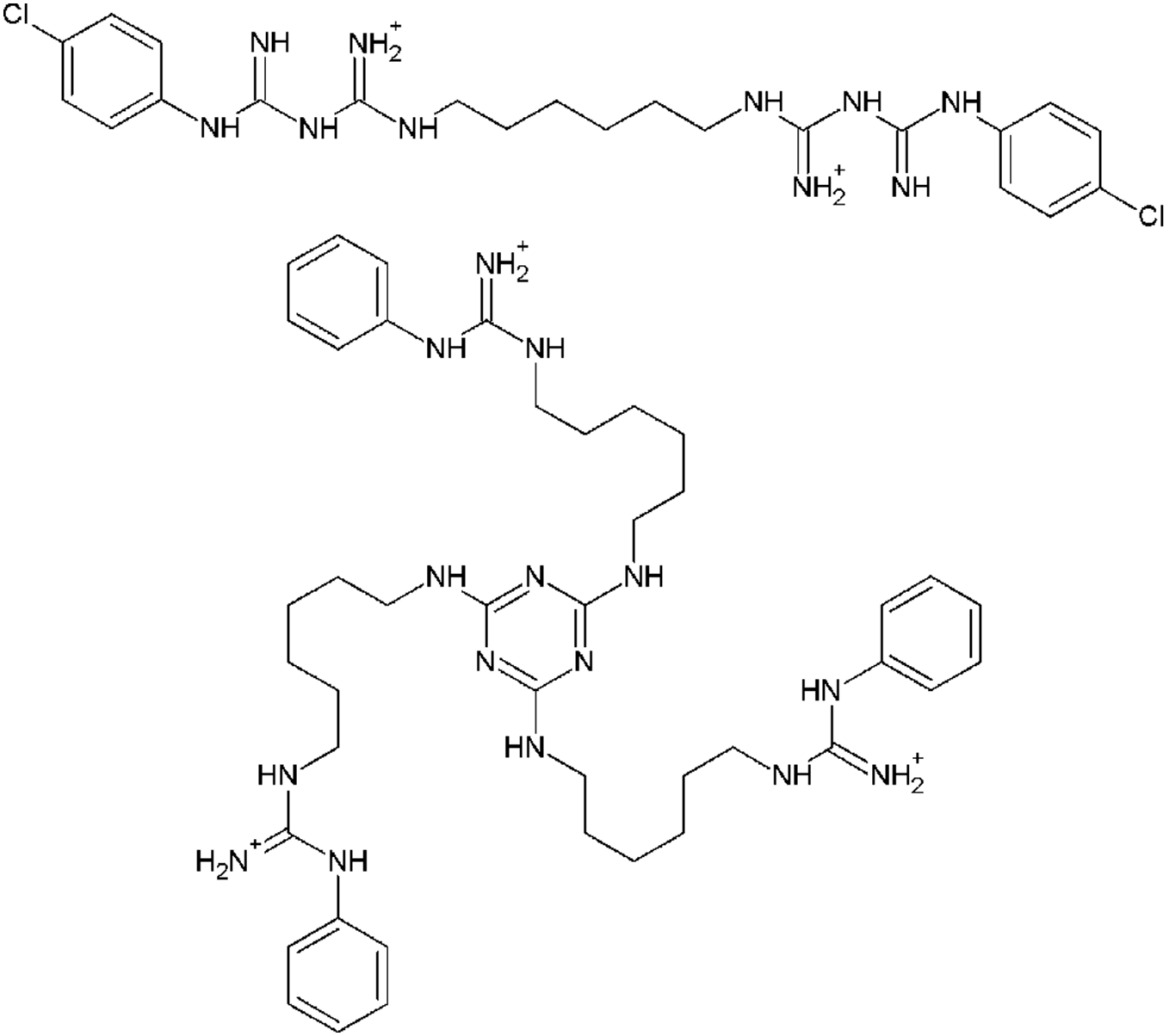
Structures of chlorhexidine (top) and THAM-3ΦG.

The most active compounds were then examined in an *in vitro* assay to determine their minimum inhibitory concentrations (MIC) against these pathogens (Table 1). Trishexylaminomelamine trisphenylguanide (THAM-3ΦG, Figure 1), which had shown antibacterial activity against almost all the strains in the viability assay, showed notable activity only against *E. coli* and *S. aureus* in the MIC assay, with some selectivity against *S. aureus* (2 mg/L) compared to *E. coli* (8 mg/L). The anti-bacterial activity against MRSA shown by THAM-3ΦG is comparable to that shown by chlorhexidine. The slightly larger TOAM-3ΦG also displayed MIC values comparable to chlorhexidine against *E. coli*, *E. faecalis,* and *S. aureus*. The derivatives of the DNT2300 and DNT2200 dendrimers showed almost no activity against any of the bacteria in the MIC assay.

**Table 1:** Minimum Inhibitory Concentrations of the most active guanide (G) and phenylguanide (ΦG) compounds.

	MIC (mg/L)							
Bacteria/Strain	*E. coli*	*P. aeruginosa*	*A. baumannii*	*A. baumannii*	*E. faecalis*	*E. faecalis*	*S. aureus*	*MRSA*
**Compound**	K91	PA01	ATCC 19606	ATCC BAA1605	MSU#10	V583	RN4220	USA300
Chlorhexidine	1	8	16	16	4	6	1	1
THAM-3ΦG	8	48	>256	>256	32	256	2	2
DNT2300-6BG	>256	>256	ND	ND	>256	ND	>256	ND
DNT2300-6ΦG	256	>256	>256	ND	>256	ND	256	256
DNT2200-8ΦG	>256	>256	ND	ND	ND	ND	>256	ND
TOAM	>64	64	>64	ND	>64	>64	>64	48
TOAM-3G	8	16	>64	ND	16	>64	2	1
TOAM-3ΦG	2	>64	>64	ND	2	4	2	2

Values are the median of at least 3 determinations. >256 indicates no inhibition at the highest concentration tested. TOAM was insoluble at >64 mg/L.

The MIC assays were repeated using Mueller-Hinton broth (MHB) for THAM-3ΦG and chlorhexidine against *E. coli* and *S. aureus* (Table 2); the results were comparable to those obtained in LB (Table 1). The minimum bactericidal concentrations (MBC) of THAM-3ΦG, and chlorhexidine were also determined in MHB for *S. aureus* and *E. coli* (Table 2). For *S. aureus*, the MBCs were similar to the MICs in each case; for *E. coli* the MBC was significantly higher than the MIC for THAM-3ΦG (Table 2).

**Table 2:** Median MICs and MBCs for chlorhexidine and THAM-3ΦG in MHB.

Bacteria/Strain	*E. coli*		*S. aureus*		*MRSA*	
	K91		RN4220		USA300	
**Compound**	MIC	MBC	MIC	MBC	MIC	MBC
Chlorhexidine	0.44	0.44	0.89	0.89	0.67	0.89
THAM-3ΦG	8.0	16	1.0	2.0	1.0	1.0

There were no significant differences between MIC and MBC for each *S. aureus* strain and no significant differences between the two *S. aureus* strains using 2-tailed unpaired T-tests (p>0.05, n≥7).

### Cytotoxicity *In vitro*

The toxicity of the compounds to human cells was evaluated using an MTS dye reduction assay in both an epithelial (MDA-MB-231) and a keratinocyte (HaCat) derived cell line (Table 3). Many of the compounds were not toxic to the human cells at the highest concentrations tested, and almost all of them were less toxic than chlorhexidine. The increased cytotoxicity (Table 3) of the TOAM compounds compared to the THAM compounds makes them less attractive as antibacterials, although the TOAM-3ΦG toxicity appeared to be no greater than that of chlorhexidine.

**Table 3:** Cytotoxicities of the guanide, biguanide, phenylguanide derivatives, and the parent amines, against a human breast cancer cell line (MDA-231) and a human keratinocyte cell line (HaCat).

Compound	CC_50_ a (mg/L)		Relative to chlorhexidine^b^	
	MDA-231	HaCat	MDA-231	HaCat
Chlorhexidine	2.6	1.9	-	-
Spermidine	22	>150	8.5	>79
Spermidine-3ΦG	430	400	170	210
Spermine	10	>200	3.7	>110
Spermine-4ΦG	>110	110	>43	56
THAM	>770	200	>290	100
THAM-3G	74	63	29	33
THAM-BG	>1000	72	>380	37
THAM-3ΦG	7.6	14	3.0	7.1
DNT2300	29	13	11	6.9
DNT2300-6G	>77	98	>30	51
DNT2300-6BG	60	11	24	5.7
DNT2300-6ΦG	280	400	110	210
DNT2200	69	16	27	8.3
DNT2200-8G	>100	710	>38	370
DNT2200-8BG	920	870	360	450
DNT2200-8ΦG	>260	210	>100	110
TOAM	ND	4.1	ND	2.1
TOAM-3G	ND	3.6	ND	1.9
TOAM-2ΦG	ND	3.1	ND	1.6
TOAM-3ΦG	ND	2.4	ND	1.3

a CC_50_ is the concentration of compound which killed 50% of the cells in the MTS assay.

b Factor by which cytotoxicity was reduced relative to chlorhexidine.

### Time-to-Kill Assays

**Figure 2:**
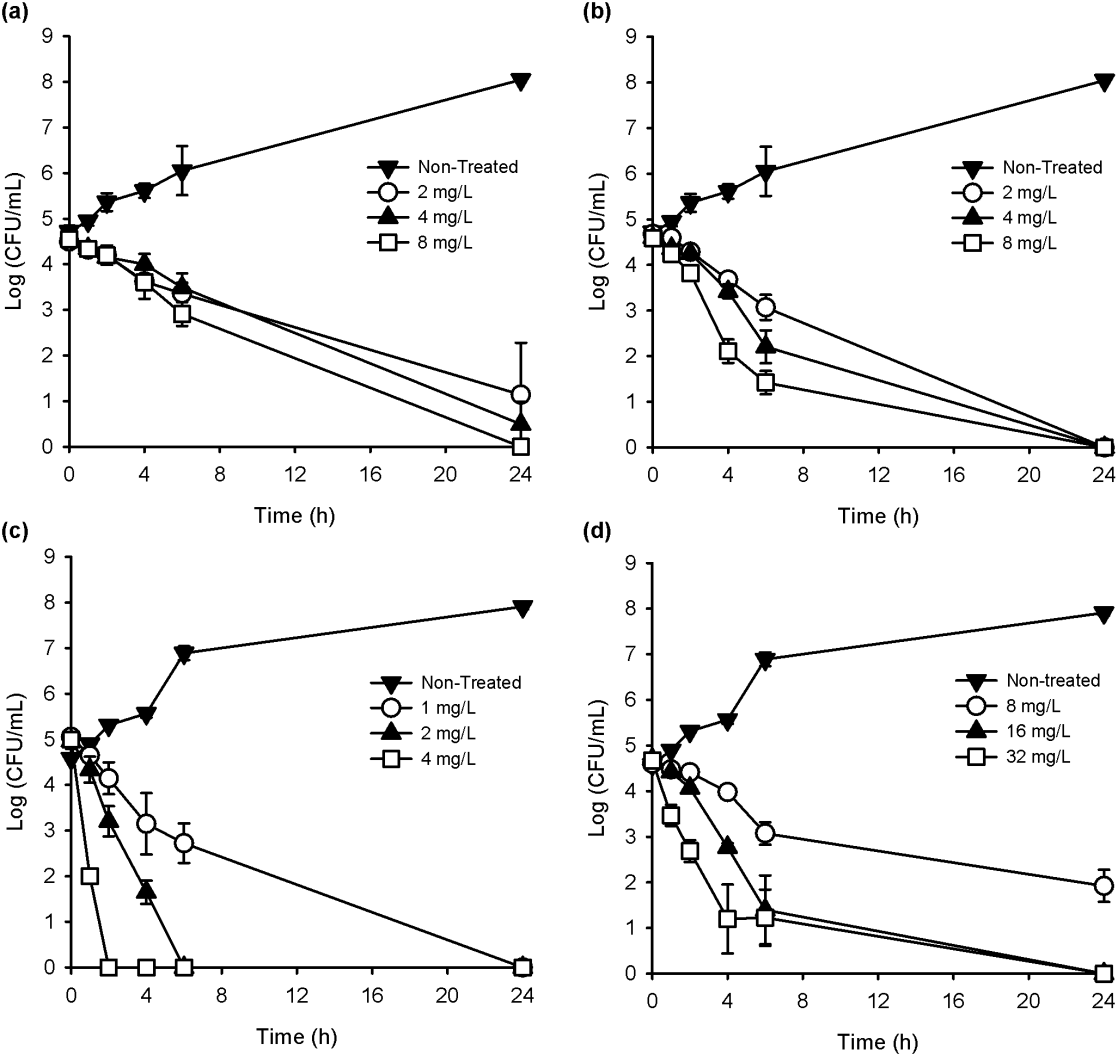
Time-to-kill assays. MRSA (a–b) and *E. coli* (c–d) treated with chlorhexidine (a, c) and THAM-3ФG (b, d). Significant differences between THAM-3ФG and chlorhexidine treatments were only seen at 4×MIC at 4 and 6 h with MRSA (p-value<0.01) and at 1 and 2 h with *E. coli* (p-value<0.05). P-values were calculated for each time point and between compounds by one-way ANOVA.

THAM-3ФG was comparable in effectiveness at killing MRSA to chlorhexidine at their MIC, and slightly more effective at 4×MIC (Figure 2). A 3-log reduction in CFU/mL was seen within 4 hours post treatment with THAM-3ФG at 4×MIC and no viable cells remained after 24 hours at the MIC. Chlorhexidine showed no significant dose-dependence of the reduction in bacterial viability over the range of 1× to 4×MIC, although bactericidal activity was seen by 24 hours. Chlorhexidine killed *E. coli* more rapidly and at lower concentrations (Figure 2a and b). At 2 mg/L it caused a >3 log reduction in CFU/mL within 4 hours of treatment, and no viable cells remained after 6 hours. At 4×MIC (32 mg/L), THAM-3ФG showed a >3 log reduction only after 4 hours of treatment, and it took 24 hours to kill all the cells at this concentration.

### Cytoplasmic Leakage Assay

**Figure 3:**
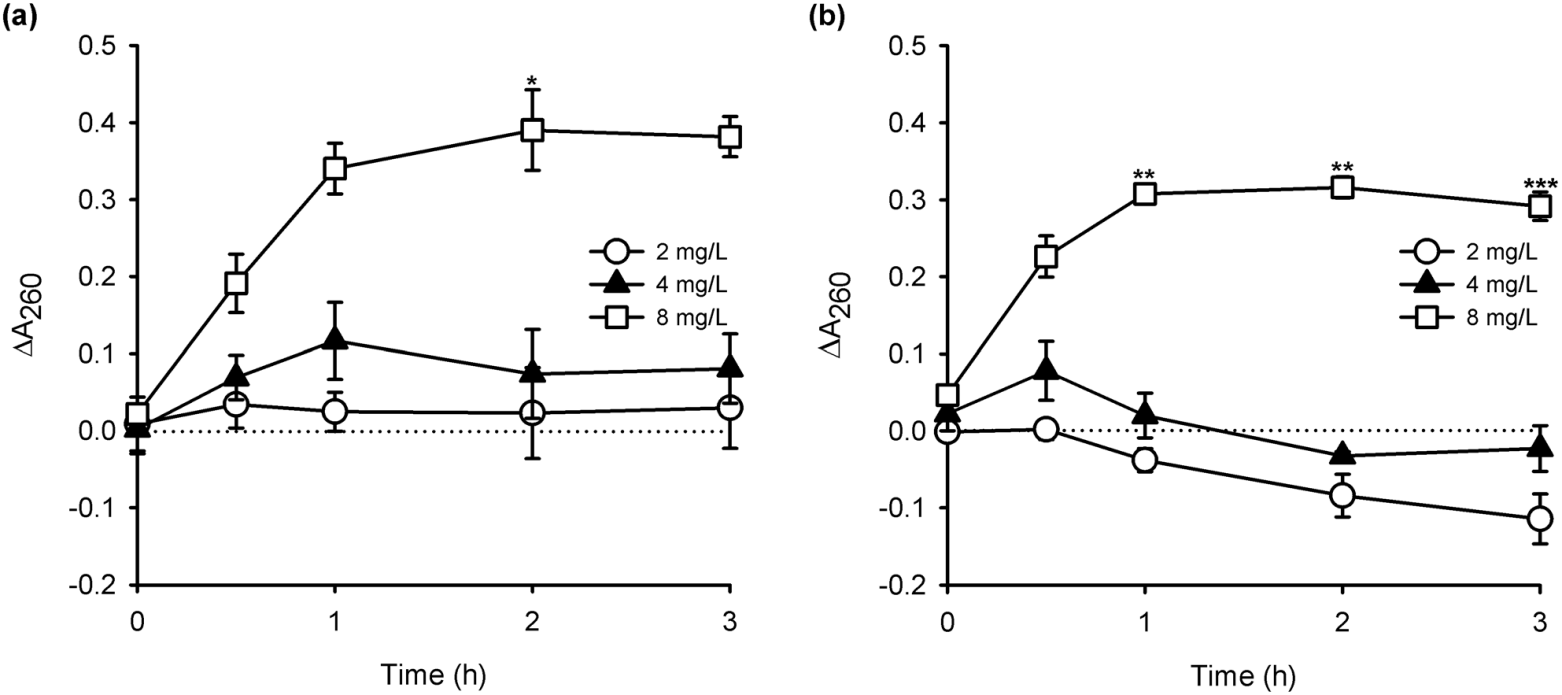
Cytoplasmic Leakage. Change in absorbance at 260(ΔA_260_) MRSA culture supernatants upon treatment with Chlorhexidine (a) and THAM-3ФG (b). Both compounds showed an immediate increase in A_260_ within a half hour after treatment at a concentration of 8 mg/L (4×MIC). Note that plots represent the difference between the absorbance of the supernatant from non-treated and treated cultures at each time point. *P-value<0.05, **p-value<0.01, and ***p-value<0.001 versus USA300 as analyzed by one-way ANOVA.

The potential for THAM-3ФG to interact with the cellular membrane of MRSA in a way that would cause leakage of cellular contents was investigated and compared to that of chlorhexidine. The cellular contents leaking out of the cell, specifically amino acids and nucleic acids, were detected by an increase in the absorbance of the cell supernatant at 260 nm over time compared to untreated controls. As shown in Figure 3, treatment with both THAM-3ФG and chlorhexidine at 4×MIC caused a significant increase in A_260_ within the first 30 minutes of treatment, as compared to untreated controls. To verify that this leakage was not secondary to cellular death resulting from other mechanisms, the number of viable cells was also determined over the 3 hour period. There was no significant reduction in CFUs upon treatment with either THAM-3ФG or chlorhexidine (Figure S1). To test the efficacy of this assay, antibiotics with known mechanisms of action that are not membrane related were also tested. Ampicillin and gentamicin, which are cell wall and protein synthesis inhibitors, respectively, showed no increase in A_260_ measurements over the same time period at four times their MIC (Figure S2). It should be noted that the cell densities used in these assays were 4 logs higher than those used in MIC determination in order to provide sufficient sensitivity for the A_260_ measurements; this may explain the slight increase in MIC seen in this assay compared to data shown in Table 1.

### MRSA Challenge

THAM-3ΦG was chosen for the *in vivo* challenge experiments, because it had the best combination of antibacterial activity and low toxicity (Tables 1–3). *In vivo* toxicity studies were carried out prior to the start of experiments in mice challenged with MRSA. Groups of three Balb/c mice each were injected *i.p.* with 0.1 ml of 0, 10, 50, and 100 µM THAM-3ФG in PBS. The highest *in vivo* dose represented 0.36 mg/kg. Blood was drawn from the mice at two hours post injection and submitted for a standard panel of serum chemistry and enzyme levels. The chemistry and enzyme levels were not elevated for any of the concentrations tested compared to the mock-treated control group (data not shown).

**Figure 4:**
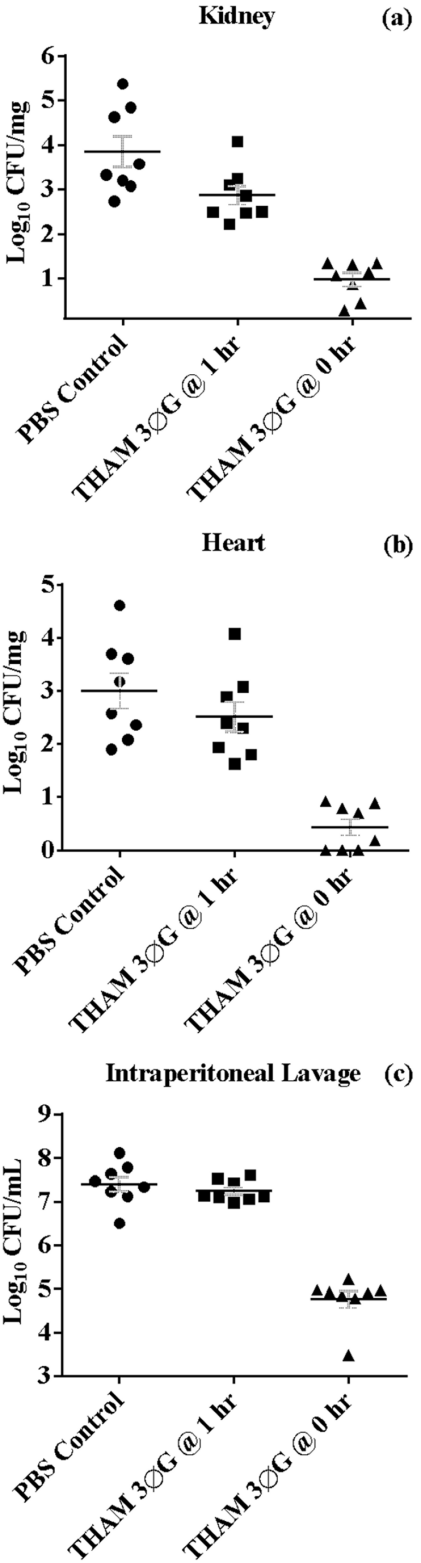
THAM-3ΦG reduces MRSA burden in vivo during peritonitis. Mice were treated with PBS control or with THAM trisphenylguanide immediately after or at one hour post intraperitoneal infection. Bacterial burden was evaluated in the kidney (A) and heart (B); localized infection was assessed with peritoneal lavage fluid (C). The horizontal bar is the mean of the eight mice per treatment group; error bars represent standard error of the mean. Reductions in burden were significant (t-test) in kidneys for both the 1 hr (p = 0.028) and 0 hr (p = 2×10^−6^) treatments. Reductions were significant in the heart (p = 4×10^−5^) and IP lavage (p = 6×10^−8^) only for the 0 hr treatments.

Since the compound was not overtly toxic, we tested its ability to reduce bacterial burden *in vivo* using a mouse model of invasive staphylococcal disease [16], [17]. Mice were treated with 100 µM THAM-3ФG either at the same time or one hour after an *i.p*. challenge with a lethal dose of MRSA. The mice treated with THAM-3ФG immediately after the MRSA challenge demonstrated significantly reduced bacterial burdens in intraperitoneal lavage, kidneys, and heart. They averaged an approximately 3-log reduction in both organs and IP lavage compared to PBS treated mice (Figure 4). Treatment of mice with THAM-3ФG one hour after MRSA infection also reduced bacterial burdens compared to untreated mice at 8 h post infection. Mean CFUs were reduced 20-fold in kidneys, 3-fold in heart and 2-fold in IP lavages (Figure 4); these values were statistically significant for the kidney samples.

## Discussion

Initial screening of our library of guanide, biguanide, and phenylguanide compounds showed that the higher molecular weight compounds had greater antibacterial activity. The derivatives of the smaller amine starting materials (1,4-butanediamine, 1,6-hexanediamine), as well as the short chain melamine derivatives (trisethylaminomelamine and trisbutylaminomelamine), and the small dendrimer PAMAM G0 were inactive at the highest concentration tested (100 µM).

A wide variation in activity vs. species of bacteria for each of the compounds was observed. The compounds ranged from being inactive against all of the bacteria tested to having antibacterial activity against both Gram-positive and Gram-negative species in the bacterial viability assays. However, none of the compounds exhibited the broad antibacterial action of chlorhexidine (MIC values ≤16 mg/L against all of the pathogens) when the antibacterial activity was tested in a complex medium (LB) against actively growing bacteria. However, THAM-3ΦG was as effective against *S. aureus* (including MRSA) as chlorhexidine and slightly less toxic to mammalian cells; this increased selectivity for MRSA gives it potential for further development.

When all the compounds in Table 1, except TOAM and its derivatives, were tested against two eukaryotic pathogens, *Candida albicans* and *Acanthamoeba castellanii,* none of them showed any substantial toxicity at 100 µM against *Candida*, or at 200 µM against *Acanthamoeba*, whereas chlorhexidine and PHMB had IC_50_ values of approximately 10 µM and 10 µg/ml, respectively, against *Acanthamoeba* (data not shown). Others have previously shown that PHMB and chlorhexidine are effective against *Acanthamoeba* [19], [20], [21], and this provides further evidence for the selectivity of THAM-3ΦG compared to the biguanides. The reduced toxicity toward eukaryotic cells is consistent with the lack of *in vivo* toxicity observed in mice. However, given the preliminary nature of these experiments, it is difficult to know how the *in vitro* toxicity compares to the *in vivo* toxicity.

The mechanism of action for the commonly used biguanide antiseptics, chlorhexidine and PHMB, is not well-defined. Although they are thought to interact with the membrane surfaces [22] resulting in leakage of cellular contents, they do not have long alkyl chains that intercalate into the lipid bilayer like those of the common antibacterial cationic quaternary amine compounds. A PHMB study [23] found induction of the *rhs* genes in *E. coli,* implicating enzymes involved in the repair/binding of nucleic acids, and another study reported cooperative binding of PHMB to nucleic acids [24]. A chlorhexidine study [25] found that the mechanism of action in *P. aeruginosa* may be multifaceted, involving changes in outer membrane permeability, the attenuation of virulence and environmental adaptation processes, and energy deprivation through the repression of genes involved in aerobic respiration. Although the fact that some of our initially screened compounds were biguanides led us to infer a similarity to PHMB and chlorhexidine, the biguanides we synthesized had little or no antibacterial activity. This observation and the differences in specificity between the phenylguanides and chlorhexidine lead us to hypothesize that THAM-3ΦG may act by somewhat different mechanisms than chlorhexidine or PHMB. Recently described compounds with multiple guanide groups, *p*-guanidinoethylcalix [4] arene [26], and Akacid plus, a mixture of polymeric guanides [27], [28], also exhibit broad spectrum activity against bacterial and eukaryotic pathogens, unlike THAM-3ΦG.

Since chlorhexidine has been observed to cause leakage of cellular contents [22], we compared its action to that of THAM-3ΦG in a cytoplasmic leakage assay. For both compounds, leakage of molecules absorbing at 260 nm was observed at concentrations that did not decrease the number of viable cells, indicating that membrane leakage precedes cell death and likely causes it at higher concentrations. In summary, THAM-3ΦG may act to disrupt bacterial membranes, but it appears to be more specific against *S. aureus* than would be predicted by such a non-specific interaction. Future studies with our compounds are aimed at further exploring its mechanisms of action.

The structural and mechanistic similarities to chlorhexidine suggested potential applications of our compounds as topical microbicides. However, quantifying effects on topical bacterial infections in vivo is difficult. Therefore, a preliminary study was done using inhibition of a systemic infection as a model for activity in a complex in vivo environment. The results from this initial in vivo antibacterial assay performed with THAM-3ΦG in mice infected with lethal doses of MRSA were promising. Reductions in bacterial load were noted when the compound was given immediately after the bacterial inoculation as well as when the compound was given one hour post infection. The smaller reduction in load seen in the latter case was likely due to the bacteria experiencing a lower exposure (concentration×time) to the compound when they had an hour to disseminate before it was administered. Although this experiment did not assess the effective dose (ED_50_), it demonstrated that the compound still exhibited antibacterial properties in a complex in vivo environment. Furthermore, the administration of only one dose at only one time point was likely sub-optimal, since the pharmacokinetics are unknown. The proposed use of these compounds is to treat skin MRSA infections; further investigation will require optimizing dosage and topical application regimens in additional studies with appropriate infection models, such as skin and soft-tissue. Nonetheless, the current study demonstrates promise for in vivo application for this compound and potentially others with related structures.

## Supporting Information

Figure S1 **Cell viability.** The number of viable cells was monitored throughout the absorbance assay. MRSA treated with Chlorhexidine (a) or THAM-3ФG (b) showed no significant reductions in CFUs.
(TIF)

Figure S2 **Cell leakage with control antibiotics.** Ampicillin (a) and gentamicin (b) were tested to confirm the efficacy of the absorbance assay as a means to monitor potential membrane disruption. Significant increases in A_260_ were not seen in either case at concentrations ranging from 1× to 4×MIC (The MICs determined using the assay described in Material and Methods were 32 mg/L for ampicillin and 0.5 mg/L for gentamicin, respectively).
(TIF)

Table S1 **Bacterial viability assays.** Bacteria were grown at 37°C to mid-log phase (OD600 ∼0.4). The bacteria were diluted to approximately 10^4^ to 10^5^ colony forming units (CFU) per milliliter and added to a solution of phosphate buffered saline (PBS, 16 mM phosphate, 0.14 M NaCl, pH 7.2) containing the compound at varied concentrations. Samples were incubated at 37°C for one hour and then plated on Luria Bertani (LB) agar at appropriate dilutions in PBS. The plates were incubated overnight at 37°C and colonies enumerated the following day. IC50s were calculated from graphs of CFU as a function of compound concentration. Chlorhexidine was used as a positive control. Included are those guanide (G), biguanide (BG), phenylguanide (ΦG) compounds that had some antibacterial activity in an initial screen at 30 and 100 µM, as well as their and parent amines. Compounds synthesized from 1,4-butanediamine, 1,6-hexanediamine, trisethylaminomelamine, trisbutylaminomelamine, and the dendrimer PAMAM (G0) were not active against *E. coli, P aeruginosa*, or *S. aureus* so they were not tested against the remaining bacteria (data not shown).
(DOC)
