# Supplementary figures and images for: Antibacterial Activity of THAM Trisphenylguanide against Methicillin-Resistant Staphylococcus aureus
Source: PLoS One. 2014 May 19;9(5):e97742. doi: 10.1371/journal.pone.0097742 (PMC4026384; doi:10.1371/journal.pone.0097742)

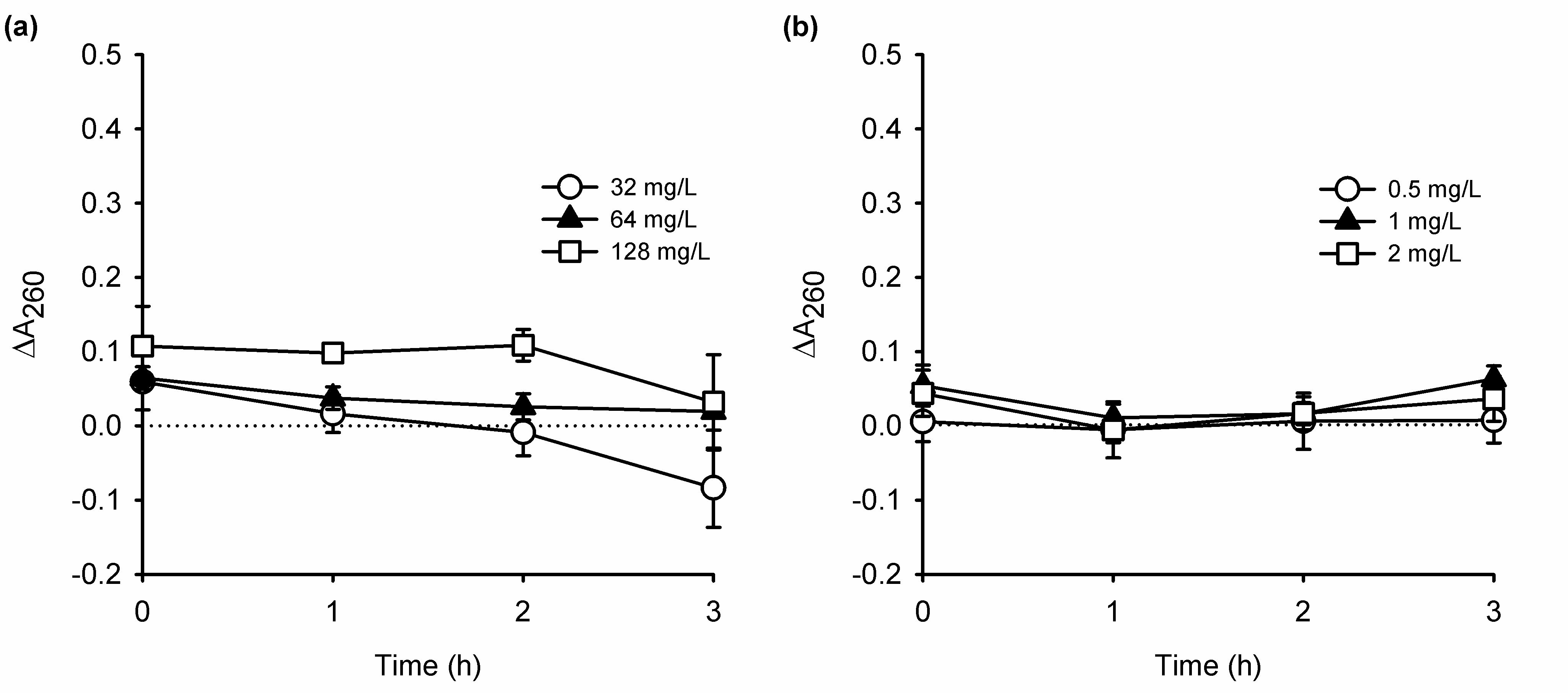

Supplement: Figure S2 — Cell leakage with control antibiotics. Ampicillin (a) and gentamicin (b) were tested to confirm the efficacy of the absorbance assay as a means to monitor potential membrane disruption. Significant increases in A260 were not seen in either case at concentrations ranging from 1× to 4×MIC (The MICs determined using the assay described in Material and Methods were 32 mg/L for ampicillin and 0.5 mg/L for gentamicin, respectively). (TIF) [file pone.0097742.s002.tif]
