# Supplementary material for: Antibacterial Activity of THAM Trisphenylguanide against Methicillin-Resistant Staphylococcus aureus
Source: PLoS One. 2014 May 19;9(5):e97742. doi: 10.1371/journal.pone.0097742 (PMC4026384; doi:10.1371/journal.pone.0097742)
Supplement: Table S1 — Bacterial viability assays. Bacteria were grown at 37°C to mid-log phase (OD600 ∼0.4). The bacteria were diluted to approximately 104 to 105 colony forming units (CFU) per milliliter and added to a solution of phosphate buffered saline (PBS, 16 mM phosphate, 0.14 M NaCl, pH 7.2) containing the compound at varied concentrations. Samples were incubated at 37°C for one hour and then plated on Luria Bertani (LB) agar at appropriate dilutions in PBS. The plates were incubated overnight at 37°C and colonies enumerated the following day. IC50s were calculated from graphs of CFU as a function of compound concentration. Chlorhexidine was used as a positive control. Included are those guanide (G), biguanide (BG), phenylguanide (ΦG) compounds that had some antibacterial activity in an initial screen at 30 and 100 µM, as well as their and parent amines. Compounds synthesized from 1,4-butanediamine, 1,6-hexanediamine, trisethylaminomelamine, trisbutylaminomelamine, and the dendrimer PAMAM (G0) were not active against E. coli, P aeruginosa, or S. aureus so they were not tested against the remaining bacteria (data not shown). (DOC) [file pone.0097742.s003.doc]

**Table S1**

Bacterial viability assays. Bacteria were grown at 37°C to mid-log phase (OD600 ~0.4). The bacteria were diluted to approximately 10^4^ to 10^5^ colony forming units (CFU) per milliliter and added to a solution of phosphate buffered saline (PBS, 16 mM phosphate, 0.14 M NaCl, pH 7.2) containing the compound at varied concentrations. Samples were incubated at 37°C for one hour and then plated on Luria Bertani (LB) agar at appropriate dilutions in PBS. The plates were incubated overnight at 37°C and colonies enumerated the following day. IC50s were calculated from graphs of CFU as a function of compound concentration. Chlorhexidine was used as a positive control. Included are those guanide (G), biguanide (BG), phenylguanide (ΦG) compounds that had some antibacterial activity in an initial screen at 30 and 100 μM, as well as their and parent amines. Compounds synthesized from 1,4-butanediamine, 1,6-hexanediamine, trisethylaminomelamine, trisbutylaminomelamine, and the dendrimer PAMAM (G0) were not active against *E. coli, P aeruginosa*, or *S. aureus* so they were not tested against the remaining bacteria (data not shown).

|  | **IC_50_ (μM)^a,b,c^** | | | | | | | |
| --- | --- | --- | --- | --- | --- | --- | --- | --- |
| **Bacteria/Strain** | ***E. coli*** K91 | ***P. aeruginosa*** PA01 | ***A. baumannii*** ATCC 19606 | ***A. baumannii*** ATCC BAA1605 | ***E. faecalis*** MSU#10 | ***E. faecalis*** V583 | ***S. aureus*** RN4220 | ***MRSA*** USA300 |
| **Compound** |
| Chlorhexidine | 1.7 ± 0.6 | 2.6 ± 0.5 | 1.3 ± 0.2 | 0.78 ± 0.31 | 14 ± 6 | 16 ± 6 | 0.86 ± 0.21 | 13 ± 0 |
| Spermidine | > 100 | > 100 | > 100 | > 100 | > 100 | > 100 | >100 | >100 |
| Spermidine-3ΦG | 3.2 ± 0.9 | > 100 | > 100 | > 100 | > 100 | > 100 | >100 | >100 |
| Spermine | > 100 | ≥ 100 | ≥ 100 | 79 ± 24 | > 100 | > 100 | >100 | >100 |
| Spermine-4ΦG | 23 ± 16 | > 100 | ≥ 100 | ≥ 100 | > 100 | > 100 | >500 | >100 |
| THAM | > 100 | > 100 | > 100 | > 100 | > 100 | > 100 | >100 | >100 |
| THAM-3G | 31 ± 12 | 14 ± 3 | 23 ± 14 | 14 ± 4 | > 100 | > 100 | >100 | >100 |
| THAM-BG | > 100 | > 100 | > 100 | > 100 | > 100 | > 100 | >100 | >100 |
| THAM-3ΦG^d^ | 3.8 ± 1.6 | 4.5 ± 2.5 | 0.86 ± 0.25 | 4.3 ± 1.0 | 2.8 ± 0.6 | 65 ± 1 | 1.7 ± 0.9 | 3.9 ± 0.5 |
| DNT2300 | 1.0 ± 0.7 | 1.4 ± 0.3 | 1.3 ± 1.1 | 4.1 ± 2.2 | 49 ± 22 | 36 ±12 | 2.1 ± 1.0 | 17 ± 3 |
| DNT2300-6G | 44 ± 1 | 8.1 ± 5.0 | 4.9 ± 1.1 | 32 ± 11 | > 100 | > 100 | >100 | >100 |
| DNT2300-6BG | 1.1 ± 0.9 | 3.6 ± 1.2 | 0.59 ± 0.36 | 1.7 ± 0.9 | 13 ± 5 | 2.2 ± 0.7 | 0.94 ± 0.14 | 1.3 ± 0.5 |
| DNT2300-6ΦG | 1.6 ± 0.2 | 5.7 ± 2.3 | 3.4 ± 1.0 | 3.6 ± 0.7 | 18 ± 15 | 17 ± 7 | 5.2 ± 1.7 | 4.7 ± 2.5 |
| DNT2200 | 5.9 ± 5.3 | 2.0 ± 1.7 | 5.2 ± 4.5 | 43 ± 17 | ≥ 100 | ≥ 100 | 4.3 ± 1.6 | 17 ± 2 |
| DNT2200-8G | ≥ 100 | 5.5 ± 0.1 | ≥ 100 | > 100 | > 100 | > 100 | >100 | >100 |
| DNT2200-8BG | 21 ± 17 | 2.3 ± 1.5 | 69 ± 31 | > 100 | > 100 | > 100 | 100 | 100 |
| DNT2200-8ΦG | 2.7 ± 0.7 | 7.0 ± 6.5 | 3.6 ± 1.9 | 5.4 ± 1.8 | 11 ± 2 | 4.0 ± 0.2 | 5.2 ± 2.0 | 3.6 ± 0.5 |
| TOAM | 3.9 ± 0.3 | > 100 | 3.9 ± 2.0 | 1.3 ± 0.6 | 77 ± 30 | > 100 | > 100 | 30 |
| TOAM-3G | 0.68 ± 0.04 | 0.85 ± 0.19 | 2.6 ± 1.1 | 0.92 ± 0.10 | 5.0 ± 1.9 | 23 ± 14 | 4.7 ± 0.1 | 1.5 ± 0.6 |
| TOAM-2ΦG | 0.75 ± 0.09 | 1.6 ± 0.4 | 0.64 ± 0.19 | 0.96 ± 0.02 | 6.1 ± 4.9 | 11 ± 3 | 2.4 ± 0.1 | 0.60 ± 0.32 |
| TOAM-3ΦG | 0.33 ± 0.11 | 1.0 ± 0.3 | 0.45 ± 0.23 | 1.7 ± 0.0 | 1.2 ± 0.1 | 1.7 ± 0.4 | 0.54 ± 0.09 | 0.24 ± 0.03 |

a. >100 means that no activity or toxicity was detected at this concentration, which was the highest tested.

b. Depending on the compound and counterion, 1μM corresponds to 0.5-2.5μg/ml.

c. The average of IC_50_ values from two separate experiments using duplicates of each compound dilution in each test. Results are in μM ± SEM.
